# Comparison of the Effectiveness, Safety, and Hemodynamic Profile of Prophylactic Administration of Carbetocin vs. Oxytocin for the Prevention of Postpartum Hemorrhage in High-Risk Cesarean Sections: A Randomized, Double-Blind Clinical Trial

**DOI:** 10.7759/cureus.89968

**Published:** 2025-08-13

**Authors:** Vrinda Oza, Kalrav Rawal, Vandana Parmar, Kiran Piparva, Ashish Chakma

**Affiliations:** 1 Anaesthesiology, Pandit Dindayal Upadhyay (PDU) Government Medical College, Rajkot, IND; 2 Pharamacology, All India Institute of Medical Sciences, Rajkot, IND

**Keywords:** carbetocin, hr, map, oxytocin, pph, uterine tone

## Abstract

Background

Postpartum hemorrhage (PPH) is a major cause of maternal morbidity and mortality, particularly in low-resource settings. Cesarean delivery increases the risk of PPH, with uterine atony being the most common cause. Oxytocin is widely used for prevention, but has limitations due to its short half-life and storage concerns. Carbetocin, a long-acting oxytocin analogue, may offer a more stable alternative.

Objective

This study’s objective was to compare the effectiveness, safety, and hemodynamic profile of prophylactic carbetocin vs. oxytocin for PPH prevention in high-risk cesarean deliveries.

Methods

The randomized, double-blind, controlled trial enrolled 70 high-risk term pregnant women undergoing elective cesarean section under spinal anesthesia. Participants were randomized to receive either carbetocin 100 µg IV bolus (group E) or oxytocin 10 IU infusion (group C - control). Uterine tone, intraoperative blood loss, additional uterotonic requirement, hemodynamic parameters, and adverse effects were assessed. An independent t-test was used for comparing quantitative variables, and the chi-square test was used for qualitative variables between the groups.

Results

The carbetocin group had significantly adequate uterine tone (Likert scores 4 vs. 3), lower need for additional uterotonics (2.86% vs. 28.57%, p = 0.0086), and reduced intraoperative blood loss (<500 mL in 82.86% vs. 42.86%, p = 0.001). The adequacy of uterine tone was highly significant (p < 0.0001) after three minutes of uterotonic administration in the carbetocin group. The carbetocin group had significantly higher mean arterial pressure (MAP) (e.g., 85.31% vs. 80% at five minutes; 89.89% vs. 82.14% at uterine repair; 91.09% vs. 86.09% at two hours) and lower heart rate (HR) (92.31% vs. 104.69% at five minutes; 90.8% vs. 101.14% at uterine repair) compared to the oxytocin group.

Conclusion

Carbetocin is a safer alternative to oxytocin for prophylactic administration to prevent PPH in high-risk cesarean deliveries with a favorable hemodynamic and safety profile.

## Introduction

Primary postpartum hemorrhage (PPH) is defined as blood loss exceeding 500 mL following vaginal delivery and 1000 mL following cesarean delivery within the first 24 hours post-delivery [1]. According to the World Health Organization (WHO), PPH accounts for approximately 27% of all maternal deaths globally, affects an estimated 14 million women each year, leading to approximately 70,000 maternal deaths annually, equivalent to one death every six minutes. The burden is disproportionately high in low- and middle-income countries (LMICs), where over 80% of PPH-related deaths occur [2]. PPH is a leading cause of maternal mortality, morbidity, and long-term complications associated with pregnancy and childbirth. However, effective preventive and treatment strategies can significantly reduce the impact of this life-threatening condition [3].

Uterine atony is the most frequent cause of primary PPH and can be prevented by the administration of prophylactic uterotonics [2]. Risk factors for uterine atony leading to PPH are uterine overdistension (e.g., multiple gestation, polyhydramnios, macrosomia), prolonged or rapid labor, chorioamnionitis, high parity, and use of uterine relaxants and uterine induction [4]. As per the systematic review and meta-analysis conducted by Yunas et al. [5], risk factors with a strong association with PPH include anemia, previous PPH, cesarean birth, female genital mutilation, sepsis, lack of antenatal care, multiple pregnancy, placenta previa, use of assisted reproductive technology, delivery of a macrosomic infant (>4500 g), and shoulder dystocia [5].

Oxytocin is the most widely used uterotonic agent with a rapid onset of action and a good safety profile. Despite being the most affordable option, oxytocin has a short half-life that necessitates repeated administration. Its utility in low-resource settings is further constrained by several additional challenges, including inadequate storage and transport infrastructure. These limitations raise concerns about product quality, heat-induced degradation, expiry, and increased wastage, all of which reduce the reliability of oxytocin in such contexts [6].

Currently, carbetocin is only licensed to be used as a prophylaxis rather than for therapeutic indications and in the context of cesarean sections. It is a long-acting, heat-stable synthetic analogue of oxytocin, described in 1987. It has a half-life of 40 minutes (around four to 10 times longer than oxytocin), and uterine contractions occur in less than two minutes after intravenous (IV) administration of an optimal dosage of 100 μg [7]. A single dose of carbetocin has been hypothesized to act as a 16-hour IV oxytocin infusion regarding the increase in uterine tone and the reduction of the risk of PPH in elective caesarean section [8]. Multiple studies suggest that prophylactic administration of carbetocin may be a promising alternative to oxytocin for the prevention of PPH in cesarean delivery, which is associated with a higher prevalence of severe PPH and requires invasive second-line therapies three times more often than vaginal deliveries do [9].

The persistence of high mortality rates from a largely manageable condition highlights the urgent need for evaluating long-acting uterotonics that improve uterine tone and hemodynamic stability and prevent PPH. The prophylactic use of carbetocin, especially in high-risk women undergoing elective caesarean delivery, and its impact on hemodynamic parameters and safety, has not been thoroughly investigated. The current study aimed to compare the effectiveness and safety of prophylactic administration of carbetocin vs. oxytocin in high-risk term pregnant women undergoing caesarean delivery.

## Materials and methods

Study design

The study was a randomized, controlled, double-blind, parallel-arm study.

Study setting

The study was conducted in the Department of Anesthesiology and the Department of Obstetrics and Gynecology at the PDU Civil Hospital, Rajkot.

Ethical consideration

This study was approved by the Institutional Ethics Committee (PDUMCR/IEC/31/2022, 30/11/2022) and registered with the Clinical Trials Registry of India (CTRI/2023/03/050426). The guidelines of this study adhere to the Declaration of Helsinki.

Study population

Inclusion Criteria

The study was conducted on 70 American Society of Anesthesiologists (ASA) physical status II and III term pregnant women aged between 18 and 40 years, at 37-40 weeks of gestation, undergoing elective cesarean delivery under spinal anesthesia (SA), with one or more risk factors for uterine atony, such as prolonged labor, uterine overdistension (e.g., multiple gestation, polyhydramnios, macrosomia), placenta previa, abruptio placenta, chorioamnionitis, or high parity, which could lead to PPH [4].

Exclusion Criteria

Term pregnant women with hypertension, preeclampsia, any contraindications to SA, need for general anesthesia, short stature, cardiac, renal, hepatic, or neuromuscular diseases, epilepsy, severe anemia, history of hypersensitivity to carbetocin/oxytocin, or who refused participation were excluded from the study.

Sample size calculation

The sample size for this study was calculated based on a pilot study conducted at our institute involving 10 full-term pregnant women, with five participants assigned to each group. The primary outcome measured was the mean uterine tone score at three minutes post-delivery, assessed using the Likert scale. The pilot study revealed a mean score of 3.8 ± 0.4 in the carbetocin group and 3.2 ± 0.5 in the oxytocin group. Based on these data, we used Zα = 1.96 (for α = 0.05) and Zβ =1.28 (for 90% power). The minimum required sample size was determined to be 30 patients per group, and considering 15% patients with insufficient data collection and dropouts, we included a minimum of 35 subjects in each group.

Blinding

The study participants and principal investigator (anesthetist) involved in the study were blinded to the uterotonic administered. The study drugs were packed in sealed, opaque envelopes to ensure blinding of the participants and investigators.

Study procedure

The study was conducted from April 2023 to May 2024 at a tertiary healthcare institute. After eligibility assessment, the written informed consent was obtained from study participants at the time of enrollment. All the term pregnant women underwent a thorough pre-anesthetic evaluation and baseline routine laboratory investigations, including coagulation profile, and liver and renal function tests. On the day of surgery, study participants were allocated into two groups by computer-generated randomized sequential number allocation, the experimental group E patients were given carbetocin (Riligol, Abbott India Ltd., Mumbai, India) 100 µg single IV bolus and control group C patients were given oxytocin 10 IU in 500 mL NaCl (0.9%) at 150 mL/hour (as per WHO recommendations) [1], over one minute on delivery of the anterior shoulder of the fetus. The term pregnant women in group C allowed a maintenance dose of oxytocin infusion (10 IU in 500 mL NaCl 0.9%) at 125 mL/hour [1]. As per the standardized anesthesia protocol, the term pregnant women were kept nil per os (NPO) for six to eight hours for solid food and two hours for clear oral fluids before being shifted to the operating room (OR). OR is equipped with all standard ASA monitors - electrocardiogram (ECG), non-invasive blood pressure (NIBP), and pulse oximeter (oxygen saturation) - which were attached in the OR. The baseline systolic blood pressure (SBP), diastolic blood pressure (DBP), mean arterial pressure (MAP), and heart rate (HR) measurements were recorded. Peripheral IV cannulas were secured in both forearms. Preloading was done with 500 mL Ringer’s lactate before SA. SA was performed at L3-L4 or L4-L5 intervertebral space with term pregnant women in the sitting position using a 25G Quincke spinal needle (Becton-Dickinson India Ltd., Chennai, India) under aseptic precautions, and 2.2 mL hyperbaric bupivacaine (0.5%) was administered. The study participants were then placed supine with a 15-degree left lateral tilt. Surgery was allowed to proceed after achieving a T6 sensory level to pinprick.

Outcome variable assessment

Prevention of PPH was evaluated in both groups using the following parameters.

Assessment of Uterine Tone

Uterine exteriorization was done, and uterine tone was assessed by the attending obstetrician at two, three, six, nine, and 12 minutes, and again at two, 12, and 24 hours post-delivery, using the Likert scale (0-4) as follows. Scores 0-2 were considered inadequate, and scores 3-4 were considered adequate for uterine tone (Table 1).

**Table 1:** Uterine tone assessment using Likert scale

Status of uterine	Score	Interpretation of score
Floppy	0	Inadequate uterine tone (scores 0-2)
Soft	1	
Poorly contracted	2	
Well-contracted	3	Adequate uterine tone (scores 3-4)
Hard rock	4	

The Requirement for Additional Uterotonics

Methyl ergometrine maleate 0.2 mg, carboprost tromethamine 0.25 mg intramuscularly, or misoprostol 200 µg orally was recorded in both groups based on the attending obstetrician’s clinical judgment.

Measurement of Intraoperative Blood Loss

Measurement of intraoperative blood loss was estimated by estimation of the quantitative blood loss (QBL) by calculating the amount of aspirated blood (suction canisters used to record volume collected before amniotomy and subtracting it from volume at the end of delivery) and the weight of surgical swabs before and after, during caesarean section [10]. The following equation was used when calculating the blood loss of a blood-soaked item: wet item gram weight - dry item weight = mL of blood within the item, assuming that 1 g of wet weight equals 1 mL of blood.

Monitoring of Hemodynamic Parameters

HR, SBP, DBP, and ECG changes were recorded at baseline, before skin incision, one, three, and five minutes after uterotonic administration, during uterine repair, and at two, 12, and 24 hours after cesarean delivery. Hypotension was defined as a decrease in MAP by 20% from baseline, and each episode was treated with IV phenylephrine or ephedrine bolus (depending on HR). Tachycardia was defined as maternal HR ≥ 120 beats per minute.

Safety Assessment

Adverse effects, either self-reported by patients or observed by the principal investigator (anesthetist), were recorded and managed accordingly.

Data analysis

Statistical analysis was performed using Epi Info 7 software (Centers for Disease Control and Prevention, Atlanta, GA). Quantitative variables were analyzed using descriptive statistics (mean, standard deviation), while categorical variables were expressed as frequencies and percentages. For outcome assessment, quantitative variables were compared between groups using the t-test or the Mann-Whitney test (for non-normally distributed data), and categorical variables were compared using the chi-square test. A p-value of less than 0.05 was considered statistically significant.

## Results

**Figure 1:**
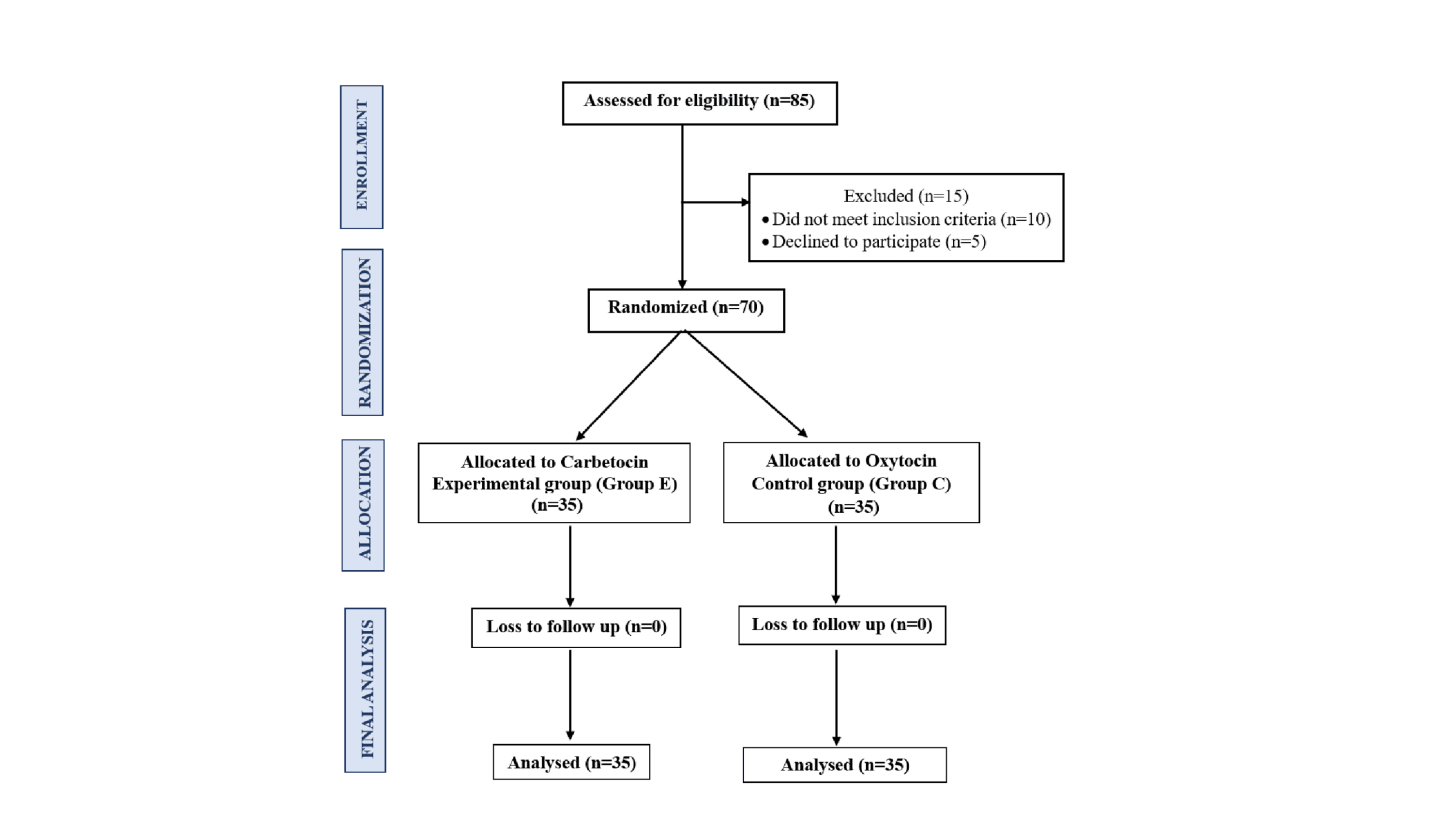
Consolidated Standards of Reporting Trials (CONSORT) diagram CONSORT diagram illustrating participant enrollment, randomization, allocation, follow-up, and analysis. Of the 85 women assessed for eligibility, 15 were excluded (10 did not meet inclusion criteria, and five declined to participate). A total of 70 participants were randomized equally into two groups: the carbetocin group (group E, n = 35) and the oxytocin group (group C, n = 35). There was no loss to follow-up, and all participants were included in the final analysis.

A total of 85 participants were assessed for eligibility. Of these, 15 were excluded - 10 did not meet the inclusion criteria, and five declined to participate. The remaining 70 eligible participants were randomized into two groups: 35 were allocated to the carbetocin (experimental group E), and 35 to the oxytocin (control group C). There was no loss to follow-up in either group. All 70 participants (35 in each group) completed the study and were included in the final analysis (Figure 1).

Table 2 demonstrates the age and gestational age of both groups, which were comparable and did not differ statistically. The mean age of participants was 27.94 ± 4.28 years in group E and 25.26 ± 5.04 years in group C. The mean gestational age at delivery was 37.94 ± 1.24 weeks in group E and 38.29 ± 0.62 weeks in group C. The most common indication for caesarean delivery in both groups was a previous caesarean section, observed in group E (65.7%) and group C (48.5%). Other indications included placenta previa (14.3% in group E vs. 22.9% in group C, non-progression of labor (8.5% vs. 17.1%), multiple pregnancy (5.7% vs. 2.9%), abruptio placenta with breech presentation (2.9% vs. 5.7%), and previous caesarean delivery with placenta previa (2.9% in both groups) (Table 2).​​​​​​​

**Table 2:** Baseline characteristics of group E and group C This table depicts the baseline characteristics of study participants in group E and group C. Data are presented as mean ± SD for continuous variables and number (percentage) for categorical variables. The age and gestational age of both groups were comparable and did not differ statistically.

Baseline characteristics	Group E (n = 35)	Group C (n = 35)
Age (years) (mean ± SD)	27.94 ± 4.28	25.26 ± 5.04
Gestational age (weeks) (mean ± SD)	37.94 ± 1.24	38.29 ± 0.62
Indication of caesarean delivery		
Previous caesarean delivery	23 (65.70%)	17 (48.50%)
Placenta previa	5 (14.30%)	8 (22.90%)
Multiple pregnancy	2 (5.7%)	1 (2.90%)
Abruptio placenta with breech presentation	1 (2.90%)	2 (5.70%)
Non-progression of labor	3 (8.50%)	6 (17.10%)
Previous cesarean delivery with placenta previa	1 (2.90%)	1 (2.90%)

**Figure 2:**
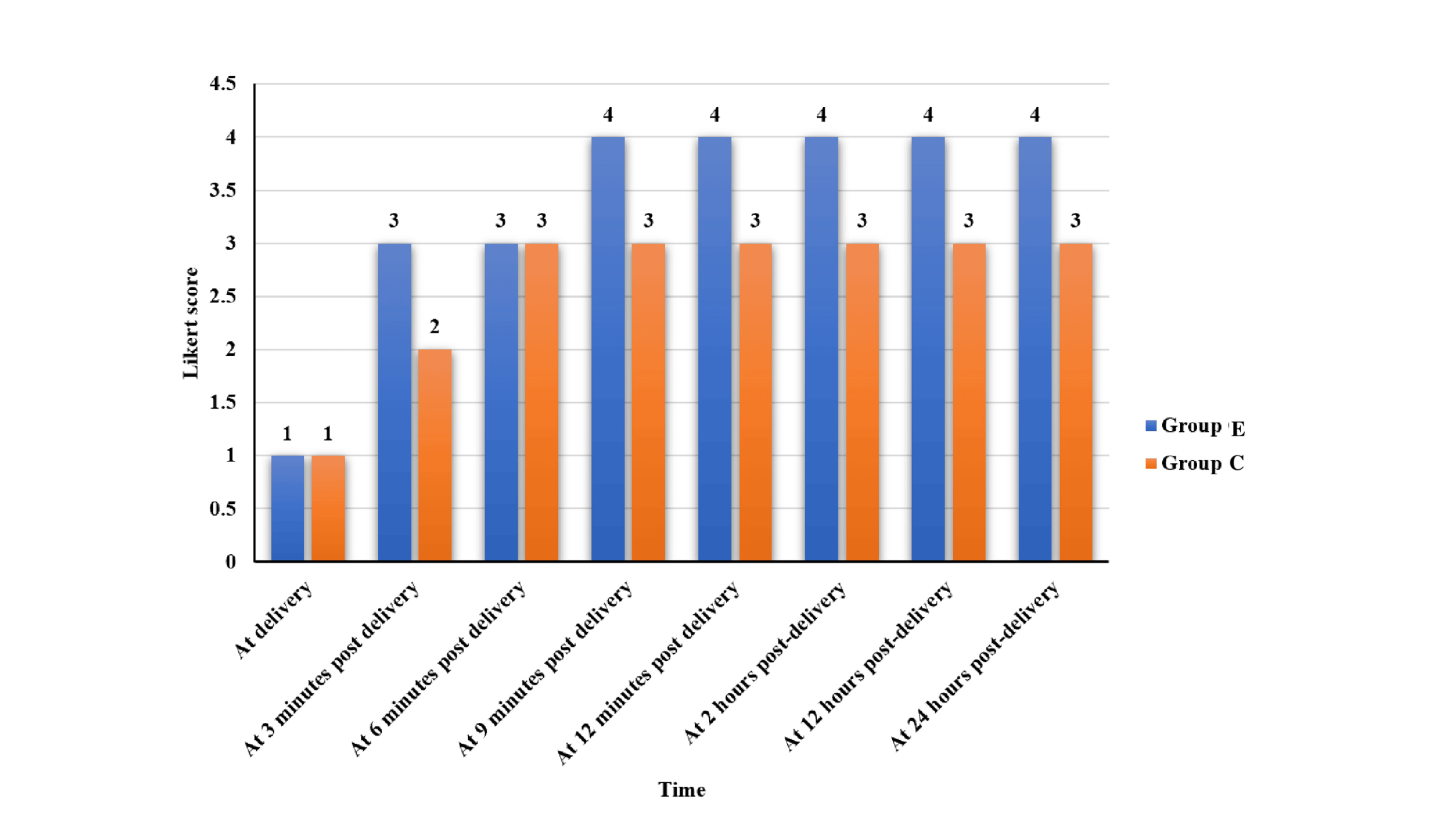
Comparison of uterine tone between group E and group C Mann-Whitney U test was used to compare the median uterine tone scores between the groups at various time intervals post-delivery, assessed using a Likert scale (1 = atonic to 5 = fully contracted). Uterine tone progressively improved over time in both groups, with consistently higher scores observed in group E, particularly between six and 24 minutes post-delivery.

Figure 2 presents the evaluation of uterine tone using a Likert scale at various time intervals (at delivery, three, six, nine, and 12 minutes post-delivery and two, 12, and 24 hours post-delivery in both group E (carbetocin) and group C (oxytocin). At the time of delivery and by three minutes of post-delivery, showed "inadequate uterine tone” Likert score less than 3). From six minutes post-delivery onward, group E consistently maintained a Likert score of 4, indicating “adequate uterine tone” throughout all subsequent assessments up to 24 hours. In contrast, group C only reached a maximum score of 3 from six minutes post-delivery onward and maintained that score at all time points, indicating persistent “inadequate uterine tone” (Figure 2).​​​​​​​

Table 3 presents a comparison of other outcome variables between group E and group C. The requirement for additional uterotonic was significantly lower in group E (2.86% vs. 28.57%, p = 0.0086) than in group C. The proportion of patients with intraoperative blood loss less than 500 mL was significantly higher in group E compared to group C (82.86% vs. 42.86%, p = 0.001). Conversely, a greater percentage of patients in group C experienced blood loss between 500 and 1000 mL compared to group E (57.14% vs. 17.14%, p = 0.001). Although the requirement for postoperative blood transfusion was higher in group C (11.43% vs. 2.86%) compared to group E, this difference was not statistically significant (p = 0.3533) (Table 3).​​​​​​​

**Table 3:** Comparison of requirement for additional uterotonics, intraoperative blood loss, and requirement for blood transfusion between group E (n = 35) and group C (n = 35) This table presents the comparison of outcome variables between group E and group C. Chi-square test (χ² test) was used to compare the need for additional uterotonic, intraoperative blood loss, and requirement of postoperative blood transfusion between group E and group C. *Significantly fewer patients in group E required additional uterotonic and experienced lower intraoperative blood loss (<500 mL) compared to group C.

Parameters	Response	Group E, n (%)	Group C, n (%)	p-value
Requirement of additional uterotonics	Yes	1(2.86%)	10 (28.57%)	0.008*
	No	34 (97.14%)	25 (71.43%)	
Intraoperative blood loss	<500 mL	29 (82.86%)	15 (42.86%)	0.001*
	500-1000 mL	6 (17.14%)	20 (57.14%)	
Requirement for postoperative blood transfusion required	Yes	1 (2.86%)	4 (11.43%)	0.353
	No	34 (97.14%)	31 (88.57%)	

Table 4 presents the comparison of hemodynamic parameters (MAP and HR) between group E and group C at delivery, one, three, and five minutes post-cesarean delivery, at the time of uterine repair, and two, 12, and 24 hours post-cesarean delivery. There were no statistically significant differences in MAP between the two groups during the preoperative baseline, before skin incision, and up to three minutes after uterotonic administration (p > 0.05). However, from five minutes post-uterotonic administration onward, significant differences were observed. At five minutes, the MAP in group E was significantly higher (85.31 ± 7.21 mmHg) compared to group C (80 ± 4.92 mmHg) (p = 0.0006). Similar significant differences were observed in group E at the time of uterine repair (p = 0.0001) and two hours after caesarean delivery (p = 0.0001). No significant difference was observed at 12 hours (p = 0.091) and 24 hours postoperatively (p = 0.42) (Table 4).

**Table 4:** Comparison of hemodynamic parameters (mean arterial blood pressure and heart rate) of group E (n = 35) and group C (n = 35) Comparison of changes in mean arterial pressure (MAP) and heart rate (HR) between group E and group C at baseline, one, three, and five minutes post-cesarean delivery, at the time of uterine repair, two, 12, and 24 hours post-cesarean delivery was performed using the unpaired t-test. *Group E exhibited significantly higher MAP at five minutes post-uterotonic administration, at the time of uterine repair, and at two hours post-cesarean delivery. **Heart rate was significantly lower in group E at five minutes after uterotonic administration and at the time of uterine repair.

Hemodynamic parameter	Time	Group E (mean ± SD)	Group C (mean ± SD)	p-value
Mean arterial pressure (MAP) (mmHg)	Preoperative baseline	94.31 ± 5.21	93.34 ± 4.81	0.42
	Before skin incision	92.71 ± 6.54	91.34 ± 5.39	0.34
	After one minute of uterotonic administration	87.37 ± 7.36	87.66 ± 4.75	0.85
	After three minutes of uterotonic administration	85.06 ± 5.68	84.66 ± 5.1	0.76
	After five minute of uterotonic administration	85.31 ± 7.21	80 ± 4.92	0.0006*
	At the time of uterine repair	89.89 ± 6.13	82.14 ± 4.36	0.0001*
	At two hours post-cesarean delivery	91.09 ± 5.01	86.09 ± 4.22	0.0001*
	At 12 hours post-cesarean delivery	91.2 ± 4.78	89.23 ± 4.87	0.091*
	At 24 hours post-cesarean delivery	94.31 ± 5.21	93.34 ± 4.81	0.42
Heart rate (HR) (beats/minute)	Preoperative baseline	92.1 ± 10.9	91.03 ± 9.26	0.66
	Before skin incision	93.6 ± 11.34	94.65 ± 10.11	0.6819
	After one minute of uterotonic administration	97.1 ± 11.67	98.63 ± 10.25	0.56
	After three minutes of uterotonic administration	101.49 ± 12.27	102.17± 11.74	0.81
	After five minutes of uterotonic administration	92.31 ± 13.29	104.69 ± 13.39	0.0002**
	At the time of uterine repair	90.8 ± 11.73	101.14 ± 14.15	0.0014**
	At two hours post-cesarean delivery	89.43 ± 10.68	94.34 ± 11.45	0.0677
	At 12 hours post-cesarean delivery	88.97 ± 9.21	89.71 ± 10.48	0.7538
	At 24 hours post-cesarean delivery	92.1 ± 10.9	91.03 ± 9.26	0.66

HR at baseline and during the early postoperative period showed no significant difference between the two groups (p > 0.05). However, at five minutes after uterotonic administration, HR was significantly higher in group C (104.69 ± 13.39 bpm) compared to group E (92.31 ± 13.29 bpm) (p = 0.0002). Similarly, at the time of uterine repair, HR remained significantly higher in group C (101.14 ± 14.15 bpm) than in group E (90.8 ± 11.73 bpm) (p = 0.0014). No significant differences in HR were observed between the groups at two, 12, or 24 hours postoperatively (Table 4).​​​​​​​

The incidence of adverse effects was significantly higher in group C (oxytocin) compared to group E (carbetocin). Higher incidence of nausea (65.71%), vomiting (37.14%), tachycardia (37.14%), hypotension (28.57%), and chest pain (68.57%) was observed in group C as compared to group E (Table 5).​​​​​​​

**Table 5:** Comparison of adverse effects between group E (n = 35) and group C (n = 35) Fisher’s exact test was used to compare the proportion of adverse events between group E and group C. Group E experienced significantly fewer adverse drug reactions, including nausea, vomiting, tachycardia, hypotension, and chest pain. Fisher’s exact test was used to compare proportions due to small cell counts.

Adverse effects	Group E n (%)	Group C n (%)	p-value
Nausea	1 (2.86%)	23 (65.71%)	0.00001
Vomiting	1 (2.86%)	13 (37.14%)	0.001
Tachycardia	3 (8.57%)	13 (37.14%)	0.01
Hypotension	1 (2.86%)	10 (28.57%)	0.008
Chest pain	0	24 (68.57%)	0.00001

## Discussion

This study assessed the prophylactic effectiveness of carbetocin compared to oxytocin in preventing PPH among high-risk term pregnant women. In this study, the carbetocin group showed a sustained effect in maintaining adequate uterine tone from six minutes post-delivery onward, compared to oxytocin, indicating its long-acting uterotonic action. This result is consistent with earlier studies, which have also demonstrated the prolonged uterotonic action of carbetocin and its potential to reduce the risk of PPH [11]. No cases of significant uterine atony leading to PPH (blood loss >1000 mL were reported in either group in our study.

The intraoperative blood loss was significantly less in the carbetocin group compared to those who received oxytocin in this study. A similar observation was reported in a study by Voon et al., that carbetocin was found to be more effective than oxytocin in reducing intraoperative blood loss and maintaining postoperative hemoglobin and hematocrit levels among women undergoing elective cesarean section [12]. As per the meta-analysis conducted by Westhoff et al., carbetocin has been shown to be effective in reducing the incidence of PPH, as well as the need for additional uterotonic agents and blood transfusion [13].

The requirement for additional uterotonics was significantly lower in the carbetocin group compared to the oxytocin group in our study. This finding is consistent with the study conducted by Kang et al., which concluded that carbetocin was more effective than oxytocin in reducing the need for additional uterotonics, while demonstrating comparable outcomes in blood loss and maternal and neonatal safety among high-risk Chinese women undergoing elective cesarean section [14]. A recent meta-analysis of five randomized controlled trials (RCTs) (N = 1214 patients) found that administration of carbetocin vs. oxytocin decreased the requirement for further use of uterotonics (odds ratio, 0.30; 95% CI, 0.11 to 0.86; I2, 90.60%) [15]. Bashir et. al. found that there was a delay in the attainment of adequate uterine tone (>1 minute) in the carbetocin group, but it sustained throughout the caesarean delivery as compared to the oxytocin group; hence, there was no need for additional uterotonics and the results at different follow-up times were consistent with our study [16]. Jaffer et al. included 46 trials and found that carbetocin was the most effective treatment in minimizing blood loss and requirement for additional uterotonics [17].

Although the requirement for postoperative blood transfusion was higher in the oxytocin group compared to the carbetocin group, the difference was not statistically significant. A similar result was reported by a study done by Fayaz et.al. comparing carbetocin and oxytocin in lower-segment caesarean section [18]. 

In our study, carbetocin was associated with significantly more stable MAP and a lower incidence of tachycardia after uterine repair, up to 24 hours. Similarly, an RCT done by Jannu et. al. observed that oxytocin produced significant tachycardia with peak effects over 180 seconds after injection and a significant decrease in MAP within 30 seconds of bolus injection and persistent hypotension, whereas carbetocin produced no significant changes in HR and MAP [19]. Similar studies have reported that carbetocin was associated with a lower rise in HR without a significant fall in MAP compared to oxytocin when used for the prevention of PPH during cesarean section [18].

In our study, the incidence of adverse effects (nausea, vomiting, tachycardia, hypotension, and chest pain) was significantly lower in the carbetocin group compared to the oxytocin group. These adverse effects may be affected by many factors like route, dose, speed of administration of uterotonics, additional uterotonics, the effect of SA, and patient factors. A study done by Khan et al. reported that a carbetocin dose of less than 60 µg was ineffective in causing adequate uterine contraction, whereas doses of more than 120 µg cause increased side effects [20].

Study limitation

The study was conducted at a single center, on a small sample size, with subjective uterine tone assessment, which may limit the generalizability of the findings.

## Conclusions

Carbetocin 100 µg single dose significantly maintained adequate uterine tone, reduced intraoperative blood loss, and minimized the need for additional uterotonics compared to oxytocin 10 IU IV infusion in high-risk parturients undergoing cesarean delivery, demonstrating a significantly better hemodynamic and adverse event profile. Prophylactic administration of a single dose of carbetocin can be a good alternative to oxytocin infusion for the prevention of PPH in high-risk term parturients undergoing cesarean delivery, offering a better hemodynamic and safety profile.
